# Picturing words? Sensorimotor cortex activation for printed words in child and adult readers

**DOI:** 10.1016/j.bandl.2014.09.009

**Published:** 2014-12

**Authors:** Tessa M. Dekker, Denis Mareschal, Mark H. Johnson, Martin I. Sereno

**Affiliations:** a Department of Visual Neuroscience, Institute of Ophthalmology, University College London, UK; b Centre for Brain and Cognitive Development, Department of Psychological Science, Birkbeck, University of London, UK; c Birkbeck-UCL Centre for Neuroimaging, Department of Psychology, University College London, UK

**Keywords:** Sensory cortex, Motor cortex, Embodiment, fMRI, Child, Reading comprehension, Animals, Utensils, Objects, Words

## Abstract

Learning to read involves associating abstract visual shapes with familiar meanings. Embodiment theories suggest that word meaning is at least partially represented in distributed sensorimotor networks in the brain (Barsalou, 2008; Pulvermueller, 2013). We explored how reading comprehension develops by tracking when and how printed words start activating these “semantic” sensorimotor representations as children learn to read. Adults and children aged 7–10 years showed clear category-specific cortical specialization for tool versus animal pictures during a one-back categorisation task. Thus, sensorimotor representations for these categories were in place at all ages. However, co-activation of these same brain regions by the visual objects’ written names was only present in adults, even though all children could read and comprehend all presented words, showed adult-like task performance, and older children were proficient readers. It thus takes years of training and expert reading skill before spontaneous processing of printed words’ sensorimotor meanings develops in childhood.

## Highlights

• We tested how picture-like responses to printed words develop in the child cortex.
• Tool versus animal pictures and their names engaged similar brain areas in adults.
• The 7–10 year-old sensorimotor cortex showed specialization for picture categories.
• But names evoked no similar BOLD patterns despite good reading in older children.
• So, automatic picturing of words’ sensorimotor meanings takes years to develop.

## 1 Introduction

Learning to read involves learning to decode the meaning of abstract word forms. Children in their early school years have a relatively good understanding of objects in the world and their labels, but are still learning to associate abstract word shapes with these familiar meanings. Embodiment theories of semantics (Barsalou, 2008, Fischer and Zwaan, 2008, Pulvermüller et al., 2005, Simmons et al., 2008) suggest that word meaning is at least partially stored in distributed sensorimotor networks across the brain, and there is now substantial neuropsychological evidence supporting these theories in adults. Therefore, to investigate how printed words become associated with word meaning as children learn to read, we investigated when and how printed word categories begin to engage the sensorimotor networks in the cortical areas activated by those categories.

In proficiently reading adults, reading a word activates the same brain regions as viewing the picture or action described by that word. For example, written tool, animal and building names engage regions in the occipito-temporal and parietal cortices of the mature brain that are also activated by pictures of tools, animals and buildings (Boronat et al., 2005, Chao et al., 1999, Devlin et al., 2005, Shinkareva et al., 2011, but see Gerlach, 2007, Tyler et al., 2003). In a seminal study, Pulvermüller et al. (2005) showed that stimulation of hand and leg areas of the left motor cortex using TMS, facilitates adults’ lexical decisions about printed arm- and leg-related words in a somatotopic manner (also see Buccino et al., 2005). Similarly, Lindemann, Stenneken, van Schie, and Bekkering (2006) showed that preparing an action involving the eyes or the mouth led to faster lexical decisions when subjects read the words “eye or “mouth” respectively. This demonstrates that sensorimotor cortex activation in mature readers plays a role in extracting meaning from printed words. Sensorimotor activations can occur rapidly and automatically in response to printed words, even when attention is distracted (Hauk et al., 2008, Kiefer et al., 2008, Shtyrov et al., 2004). They are also, however, modulated by task context (Hoenig et al., 2008, Simmons et al., 2008). For example, BOLD responses in the adult brain are more pronounced during tasks involving deliberate retrieval of category-specific object features than during tasks that do not, such as purely perceptual tasks (e.g., size discrimination), or name or function retrieval (Boronat et al., 2005, Devlin et al., 2005, Kellenbach et al., 2003, Noppeney et al., 2006, Tomasino et al., 2007). Sato, Mengarelli, Riggio, Gallese, and Buccino (2008), found that reading hand-action verbs only interfered with manual button presses during an explicit semantic judgment task, and not during lexical decision-making. Together, these findings demonstrate a strong functional coupling between visual word form areas and sensorimotor representations in the cortex of proficient adult readers. While it is still unclear how flexibly these distributed cortical networks contribute to semantic processing across different task contexts (Mahon and Caramazza, 2008, Pulvermueller, 2013, Willems and Casasanto, 2011), evidence suggests convincingly that sensorimotor activation in response to printed words reflects semantic processing.

In UK primary schools, children learn to read simple words during their first year when they are 4–5 years old. Reading fluency continues to develop substantially after that, with improvements in reading speed and accuracy extending until around the 15th year of life (Wechlser, 2001). Age-related changes in reading skills are accompanied by focalisation and left-lateralisation of word shape selective occipito-temporal areas (Brown et al., 2005, Schlaggar and McCandliss, 2007, Schlaggar et al., 2002) and decreasing activation in posterior temporal areas associated with cross-modal orthographic and phonological processing (Church et al., 2008, Pugh et al., 2001). While substantial research has charted how structural and functional changes in these language-related areas contribute to reading improvement during development, the role of cortical sensorimotor representations in this process has not yet been explored. It is therefore unclear when printed words start engaging the same brain areas as their pictorial counterparts as children learn to decode meaning from word forms. Understanding this process can provide important insight into how and under which circumstances child readers access the sensorimotor meaning of written words, and provide a benchmark for investigating word comprehension in children with reading difficulties. This research can also inform theories on how distributed semantic sensorimotor networks contribute to the printed word-learning process.

Only a few studies have investigated distributed semantic networks in the developing sensorimotor cortex, but initial evidence suggests that these might already be present before children learn to read. For instance, by 6–7 years of age, passive viewing of tool pictures without the overt plan to act, engages grasp-related areas of the cortex whilst passive viewing of animal pictures does not (Dekker, Mareschal, Sereno, & Johnson, 2011). Similarly, by 4 years of age, listening to actions words (verbs) activates motor areas in the brain, but listening to non-action words (nouns) does not (James & Maouene, 2009). Which role might such already-established cortical sensorimotor representations play during reading acquisition? It is possible that sensorimotor networks become involved early during reading training, for example because they may help bootstrap the formation of mappings between word shape and word meaning (Nation, 2008). Or, underdeveloped spelling/sound connections might allow for a greater influence of semantic information on slow word-recognition processes (Plaut & Booth, 2000). In line with an early role for sensorimotor representations in word comprehension several classic studies have shown that the semantic relatedness of task-irrelevant words embedded in pictures influenced picture naming speed to similar extents in adults and 7 to 8-year-old school children (Ehri, 1976, Rosinski, 1977, Rosinski et al., 1975). This suggests that there were shared representations for printed word forms and their corresponding pictures in both groups. Initial TMS studies show that in adults, the motor cortex plays a functional role in word-to-word priming effects on tools (Cattaneo et al., 2010, Tremblay et al., 2012). It is unclear whether similar mechanism give rise to picture-word priming effects (Mahon et al., 2007, Mulatti and Coltheart, 2012), but this seems a plausible possibility. Based on early development of picture-word priming effects, we might thus expect that printed words automatically engage similar brain areas as the pictures they describe from the 7th year of life onwards, when children have just learnt to decode basic written word meanings.

To test this hypothesis, we characterised the emergence of picture-like BOLD responses for single printed utensil (tool) and animal names in children aged 7–11 years and adulthood. This age range allowed us to include children who had already acquired the printed words in the experiment but who showed substantial differences in reading skill and age. Tool and animal stimulus categories were selected because in subjects of all ages in the experiment, tool and animal pictures activate distinct cortical sensory and motor regions. These category-selective activations overlap with brain areas that process prominent category features; Enhanced responses for tools versus animals (tool selectivity) are found in areas associated with grasping, reaching, tool motion and object shape, while enhanced responses for animals versus tools (animal selectivity) is present in low-level visual areas and – albeit less so for children – in areas associated with face and body perception (Chao et al., 1999, Dekker et al., 2011, Johnson-Frey, 2004, Lewis, 2006). With the possible exception of low-level visual areas, these are not purely sensory or motor regions. Electrophysiological recordings reveal that several tool-selective areas contain mixtures of visual, motor, visuomotor and other types of uni-and multisensory neurons (Arbib, 2008, Graziano and Gross, 1998, Murata et al., 2000), and in various regions tool and animal selective representations can be activated by multiple senses (Mahon et al., 2009, Peelen et al., 2014, Striem-Amit and Amedi, 2014). Whilst neural representations within these areas are multisensory in nature and hence arguably more “abstract” than neural representations in the primary visual and motor cortex, we will refer to them as sensorimotor areas for simplicity.

During the fMRI experiment in the scanner, children and adults viewed blocks of tool pictures, animal pictures, the corresponding printed tool and animal names and a fixation baseline. We were particularly interested in when and how spontaneous sensorimotor responses to words develop in the cortex (see hypothesis). Therefore we employed a one-back basic-level object categorisation task without explicit instructions for object property retrieval. In this task, subjects pressed a button when the same basic-level category picture or name was presented twice successively. Effects of category-changes (tools versus animals) on the BOLD signal were measured for different stimulus formats (word versus picture) and compared across age.

## 2 Materials and methods

### 2.1 Subjects

Thirteen adults (average adult age = 28.1, SD = 5.4, range 23–45 years, 5 males), and twenty-one 7- to 10-year-olds took part in the study. Children were split into two groups with eleven 7 to 8-year-olds (average age: 7.6, SD = 0.41, 7 males) and ten 9 to 10-year-olds (average age: 9.8, SD = 0.41, 8 males). One additional child was excluded due to exceptionally poor task performance, and two for failing to match all words in the experiment to their corresponding picture. Five additional children were excluded because they moved more than 2 mm in total (>57% of a voxel) during three or four runs. This strict maximum movement criterion was chosen to limit motion-induced noise in paediatric data relative to adult data. Additional analyses were performed on the remaining data to further reduce any effects of motion artefacts (see Section 2.5.2 in Methods and materials). All participants were neurologically normal, right-handed with normal or corrected vision. Research was executed under approved University protocols for human adult and minor participants in research.

### 2.2 Stimuli

**Fig. 1:**
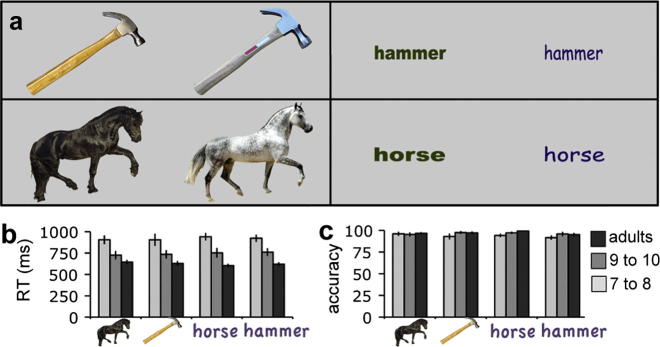
(A) Stimuli for fMRI were animal pictures, tool pictures, animal words and tool words. Participants pressed a button when the same basic-level category member was presented twice in a row. (B) Accuracy and (C) response times on the one-back task during fMRI. Dark grey: adults, medium grey: 9- to 10-year-olds, light grey: 7- to 8-year-olds.

fMRI stimuli were colour photographs and written names of 20 types of familiar tools and animals (see Fig. 1A) presented against a light grey background. There were two exemplars per item, which varied in colour, size, area on the screen, and shape- or font in the case of printed names. Crucially, as a result of these variations, the task could not be solved by a direct visual matching strategy. To ensure that the visual properties of printed names were as similar as possible across categories, each tool word was visually matched to an animal word. Images were projected onto a back-projection screen at 97 cm distance (23 × 14° visual angle, screen resolution 800 × 600) via a double mirror, using Matlab 6.0 (Mathworks) and Cogent 2000 programs. Pictures were fit to a centred 600 × 450 pixel rectangle, and words to 400 × 120 pixels. Tool and animal words were matched on average number of letters, syllables and written word (British version of Celex2 database, (Baayen, Piepenbrock, & Gulikers, 1995, see Appendix A. Table 1). Words were also matched across category for size, location, colour and font. A black-outlined red fixation cross was displayed for all pictures (but not words), during fixation blocks and inter-stimulus intervals.

### 2.3 Procedure

Before the fMRI experiment participants were asked to match word stimulus cards to picture stimulus cards. Those who failed to match all stimuli were excluded from the study (2 7-year-olds). Reading fluency for experimental words was measured outside the scanner in a self-paced reading-words-aloud task. Reading accuracy and the time from word presentation to next word-initiating button press were recorded. In the scanner, children received movement reduction training whilst watching a funny cartoon. The cartoon was paused when an MR-compatible video camera recorded excessive movement. This training continued until the participant was lying sufficiently still for several minutes.

During the fMRI experiment, participants performed a one-back categorisation task; they pressed a button with their right index finger when the same animal or tool picture (e.g., white cat, black cat) or the same animal or tool word (e.g., ***CAT***, cat) was presented twice in a row. Each trial began with a 1.5 s stimulus followed by a 0.8 s fixation screen. With this presentation duration, it is highly unlikely that subjects of any age failed to process word content, since from age 7 years onwards, semantic priming effects occur for briefly presented words (Chapman et al., 1994, Plaut and Booth, 2000), even when word primes are task irrelevant (Simpson and Foster, 1986, Simpson and Lorsbach, 1983) or ignored (Ehri, 1976, Rosinski et al., 1975). Responses were recorded with a Lumitouch button box. Participants were instructed to fixate a central cross at all times, except during word blocks, when the cross was not present.

There were 4 runs of 6 min 42 s. Each run consisted of 5 animal picture blocks, 5 tool picture blocks, 5 animal word blocks, 5 tool word blocks and 5 fixation baseline blocks of 16.1 s each (7 trials). Block and stimulus order were randomised with no stimulus repetitions within blocks. Target trials occurred 12 times during each run – 3 times for each stimulus category. Button-press-related motor activation in the brain should not affect any contrasts of interest because (a) responses were infrequent, and (b) matched across conditions. To keep participants motivated, hits and false alarms were shown after each run. After fMRI, children’s reading abilities were measured using the Sight Word Efficiency Subtest of the TOWRE (Torgesen, Wagner, & Rashotte, 1999), a standardized test of reading accuracy and efficiency for pronouncing printed words. Raw scores reflect the number of words on a list that are read accurately within 45 s.

### 2.4 MRI parameters

MR data were collected with a Siemens TIM Avanto 1.5T scanner, using a 32-channel receive-only head coil. Data from 5 adults was collected without the front part of the coil (leaving 2/3 of the channels). Because this only leads to a lower signal to noise ratio in the orbitofrontal regions it did not affect any regions where an effect was expected, and so the data of these participants was included in the analysis. A high-resolution T1-weighted 3D MPRAGE T1-weighted structural scan (1 × 1 × 1 mm voxel size, bandwidth: 190 Hz/pix, image matrix = 224 × 256, 176 partitions, TR: 2730, TE: 3.57, effective TI 1000 ms, flip angle: 7 degrees) was used for alignment. Functional runs were collected using single-shot EPI (32 slices, 164 volumes, axial plane, interleaved, bandwidth = 1906 Hz/pix, matrix 64 × 64, TR: 2.5 s, TE: 39 ms, flip: 90 deg, voxel size: 3.5 cm^3^).

### 2.5 fMRI Analysis

After discarding the first 4 volumes, the times series was registered using FSL MCFLIRT, (Jenkinson, 2002). A 5 mm FWHM Gaussian smoothing kernel was applied, and the data were temporally high-pass filtered to remove linear trends. After brain structures were removed with FSL’s Brain Extraction Tool (Smith, 2002), functional images were registered to the T1 weighted 3D MPRAGE that was aligned with the Montreal Neurological Institute Talairach compatible MR atlas of 152 averaged adult subjects using FSL FLIRT. By 6 years of age, brain volume reaches 95% of its peak size (Lenroot & Giedd, 2006). Alignment of child brains to an adult template after this age has been validated by several studies revealing negligible differences in anatomical loci and functional activation peaks of adults and children aged 7 years and older (Burgund et al., 2002, Kang et al., 2003, Muzik et al., 2000).

Positive excursions, and undershoots in the hemodynamic response were accounted for by convolving the events of each condition with a double-gamma basis function. The temporal and spatial derivatives of the hemodynamic response function were also added, to account for variations in the shape and time course of the hemodynamic response across brain regions and individuals. Only runs with less than 2 mm absolute movement were included (included number of runs: 7- to 8-year-olds = 25, 9- to 10-year-olds = 33, adults = 52). Regressors of interest for animal picture-, tool picture-, animal word-, and tool word- presentation times were created for runs that met the inclusion criteria for motion artifacts. Motion artifacts may remain after standard motion correction procedures for large scan-to-scan movements (Diedrichsen & Shadmehr, 2005). We therefore created additional regressors of non-interest for each scan that had translated half a millimeter or rotated one degree or more with respect to the previous one. Because motor responses were infrequent and matched across conditions, target trials were not modelled in the design matrix. This is the convention for one-back tasks in fMRI (Golarai et al., 2007, Tong et al., 2000, Williams et al., 2004, Yovel and Kanwisher, 2004). Degrees of freedom estimates were corrected for autocorrelation in the time course using FSL pre-whitening (Woolrich et al., 2001). Individual runs were combined at the second level in a fixed effects analysis to obtain cross-run averages. At the group level, random-effects components of mixed effects variance were modelled and estimated for each contrast (FLAME, Beckmann et al., 2003). To identify significant clusters of activation, *Z*-statistic images (Gaussianised T/F) were thresholded *z* = 2.3, *p* = 0.01 at voxel level, and a cluster size probability of *p* < 0.05. Identifying sensorimotor activation in response to printed words often requires the increased power of region of interest (ROI) analyses (Willems & Casasanto, 2011). Therefore, two complementary ROI analyses were performed in addition to a whole brain analysis.

#### 2.5.1 ROI selection

##### 2.5.1.1 ROIs derived from group average activation maps

In a first set of ROI analyses, group average ROIs were derived from significant tool or animal category-specific clusters within each age group’s average activation map. For each individual within the group, mean BOLD responses to tool and animal words and pictures were then extracted from these group-specific ROIs. The advantage of this selection procedure is that it allows for straightforward identification of age-appropriate ROIs. A limitations of this approach, however, is that category selective responses underlying mean activations may be more variable at younger ages, so average activation clusters may be less representative of individual activation patterns in earlier childhood (Poldrack, 2010). In addition, due to thresholding, different combinations of tool- and animal selective areas are grouped into single ROI clusters in different age groups, rendering comparisons across age for a given tool or animal region difficult to interpret. To account for these factors, an additional set of ROIs was defined consisting of category-selective voxels in pre-defined cortical regions within the individual activation maps.

##### 2.5.1.2 ROIs derived from individual activation maps

To select cortical areas with category-selective voxels in each individual activation map, we first created eight large spherical volumes (15 mm diameter) centred on average peak voxels or centre of gravity coordinates of tool- or animal selective areas reported in the literature. The spheres were located in the tool picture selective left AIP (*x* = −44, *y* = −37, *z* = 44), left IFG (*x* = −46, *y* = 13, *z* = 14) left LOC/MTG (*x* = −48, *y* = −60, *z* = −4.1) (Valyear, Cavina-Pratesi, Stiglick, & Culham, 2007) and the left and right medial FFG *x* = −25, *y* = −57, *z* = −7 and *x* = 22, *y* = −57, *z* = −5 (Chao et al., 1999, Devlin et al., 2005), and in the animal picture selective left and right lateral FFG: *x* = −38, *y* = −58, *z* = −12 and *x* = 36, *y* = −58, *z* = −12 (Chao et al., 1999, Devlin et al., 2005) and right posterior LOC, *x* = 46, *y* = −70, *z* = −1 (Grill-Spector, Knouf, & Kanwisher, 2004; Peelen & Downing, 2005). Crucially, previous findings (Dekker et al., 2011) corroborated by the current results, suggest that the overall organisation of tool and animal-selective areas across the brain is qualitatively adult-like by 6 years of age, and hence that identifying tool and animal picture-selective voxels of adults and children in the same cortical regions, is appropriate in this case. Nevertheless, the spherical ROIs where kept large, to account for any age-related variability in tool and animal selective peak-activations and the distribution of active voxels around these peaks. Subject-specific voxels of interest were defined by identifying all animal and tool picture selective voxels (*p* = 0.05, uncorrected) within each sphere for each individual. Finally, the BOLD-response to animal and tool words were extracted from these voxels and compared across age.

#### 2.5.2 Control analyses

Higher BOLD-related confounds in children can compromise the results of age-comparisons. As described in the previous section, harmful effects of motion artefacts were minimised by applying strict run exclusion criteria for overall motion, and by capturing signal changes resulting from small sudden movements in regressors of non-interest. To exclude the possibility that despite these procedures, age-differences in picture-like responses to printed words could still be driven by larger BOLD-related confounds in children, we tested if age differences across all subjects persisted when the same comparisons were performed across sub-groups of adults and children matched on the following two noise indices:

##### 2.5.2.1 Motion

Because sudden movements can leave residual noise in the BOLD-signal after registration, scan-to-scan motion is a good indicator of motion-related variance in the signal after standard correction procedures are applied. The mean Euclidian translational movement distance Δ*D* from one volume to the next was calculated in millimetres and the mean absolute scan-to-scan rotational motion Δ*θ* was calculated in radians:

$$\Delta D=\frac{\sum_{\mathrm{TR}=1}^{N-1}\sqrt{{\left(X_{N+1}+X_{N}\right)}^{2}+{\left(Y_{N+1}+Y_{N}\right)}^{2}+{\left(Z_{N+1}+Z_{N}\right)}^{2}}}{N-1}$$

$$\Delta \theta =\frac{\sum_{\mathrm{TR}=1}^{N-1}\sqrt{\text{abs}({\text{pitch}}_{N+1}+{\text{pitch}}_{N})+\mathrm{abs}({\mathrm{roll}}_{N+1}+{\mathrm{roll}}_{N})+\mathrm{abs}({\mathrm{yaw}}_{N+1}+{\mathrm{yaw}}_{N})}}{N-1}$$

##### 2.5.2.2 Residual error of the GLM (%Res)

This reflects residual variance in the data unaccounted for after fitting the full General Linear Model with regressors of interest and nuisance regressors. It is an inclusive measure of BOLD-related noise and goodness of model fit. For animal and tool picture category-selective voxels in each spherical region of interest, residual variance of the GLM was extracted from the subject/scan.feat/stats/sigma-squaredes.nii images in FSL that were first resampled to standard space and averaged across all scans. Using the formula reported in (Golarai et al., 2007), we then computed mean percentage of residual noise in the signal of each ROI:

$$\%\text{Res}=100\times \frac{\frac{1}{\mathrm{Nvox}}\sqrt{\sum_{i=1}^{\mathrm{Nvox}}}\mathrm{Sigmasquareds}(i)}{\mathrm{Mean}\;\mathrm{Amp}}$$

*Mean Amp* is the average BOLD signal across all scans within the relevant voxels of interest, extracted from the mean_func.nii.gz image in the second-level subject/allscans.gfeat folder in FSL. Finally, resulting %Res values were averaged across all ROIs to obtain one total value per subject.

In the Appendix B, Table 1, these indices of noise in the data are reported for all age groups, and for two subgroups of 9 adults and 9 children matched on these BOLD-related confounds. Control analyses with these matched sub-groups are reported in the final section of Section 3.

## 3 Results

### 3.1 Behavioural performance in the scanner

Accuracy on the one-back basic-level categorisation task in the scanner was “high” (>85%) at all ages and across all stimulus categories (animal pictures, tool pictures, animal words, tool words; see Fig. 1B). There were no significant over-all effects of Category (*F*(1, 31) = 0.941, *p* = 0.340), Format (*F*(1, 31) = 0.0289, *p* = 0.595), nor any interaction between Category × Format (*F*(1, 31)=1.350, *p* = 0.254). Performance was equivalent at all ages; there was no main effect of Age: *F*(2, 31) = 2.2, *p* = 0.13, no interaction of Age × Category (*F*(2, 31) = 0.436, *p* = 0.650), Age × Format (*F*(2, 31) = 0.021, *p* = 0.811), nor a 3-way Age × Category × Format interaction (*F*(2, 31) = 0.510, *p* = 0.606). Response times did not depend on Category (*F*(1, 31) = 0.011, *p* = 0.916), Presentation mode (*F*(1, 31) = 0.286, *p* = 0.596) or an interaction between these factors (*F*(1, 31) = 0.037, *p* = 0.849). Response times decreased with age (*F*(2, 31) = 17.63, *p* < 0.001; see Fig. 1C) but this decrease was not modulated by Category or Format (Category × Age (*F*(2, 31) = 0.262, *p* = 0.771); Format × Age (*F*(2, 31) = 0.780, *p* = 0.467); Category × Format × Age (*F*(2, 31) = 0.355, *p* = 0.704). Hence, any age-related differences in category-dependent neural responses to pictures or words cannot simply be attributed to differences in task performance.

### 3.2 Behavioural measures of reading ability

Before the experiment we ensured that all subjects could match each animal and tool name in the stimulus set to its appropriate picture, such that even the youngest children were able to read and understand the meaning of all words in the scanner. A computerised, self-paced reading task outside the scanner revealed that reading accuracy was high for the words in the experiment for each of three age groups (7- to 8-year-olds: 97% correct (SD = 0.03), 9- to 10-year-olds: 99% correct, (SD = 0.01), adults: all 100% correct). It is important to note that even in this self-paced task in which subjects could take breaks, the average time it took to pronounce a word and initiate presentation of the next one by pressing space was considerably shorter than the stimulus presentation time in the scanner (presentation time in scanner: 1.5 s, longest average reading time: 1.28 s). A standardized printed word pronunciation test (the Sight Word Efficiency Subtest of the TOWRE; (Torgesen et al., 1999), revealed that reading fluency improved substantially between age 7 and 10 years, with raw scores of 53.5 (SD = 13.7) at 7–8 years and 72.6 (SD = 6.5) at 9–10 years. TOWRE norms for adults are established at 98, (SD = 14), less than 2 standard deviations above the mean score of 9 to 10-year-olds. Indeed, the older children reported reading books such as *Harry Potter* in their spare time. In sum, all children in the study could read and comprehend the words in the experimental set, and the older children possessed good, close-to-adult-like reading fluency.

### 3.3 MRI analyses

#### 3.3.1 Whole brain analysis

##### 3.3.1.1 Responses to tool and animal pictures per age group

**Fig. 2:**
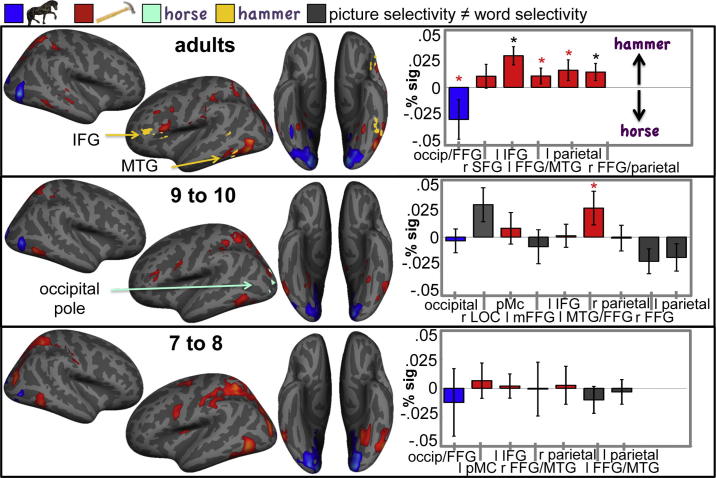
Left: Group average activation maps of animal picture selective regions (blue), tool picture selective regions (red), animal word selective regions (light green) and tool word selective regions (yellow) at age 7–8, 9–10 and adulthood. Maps are displayed on the average Freesurfer white matter surface. Activation was thresholded at *z* = 2.3, *p*_cluster_ < 0.05. Right: Category preference for tool vs animal words extracted from group tool picture or animal picture-selective clusters in adults, 9- to 10-year-olds and 7- to 8-year-olds displayed in top panel. Blue bars indicate animal picture selective ROIs with a corresponding preference for animal words. Red bars indicate tool picture selective ROIs with a corresponding preference for tool words. Grey bars reflect ROIs with an inconsistent category preference for pictures and words. Significant category preferences for words are indicated by black stars (*p* < 0.05, one-tailed *t*-tests), and trends by red stars (*p* < 0.1, one-tailed *t*-tests).

Cortical areas with a preference for tool or animal pictures were defined as a set of contiguous voxels where (tool pictures–fixation) > (animal pictures–fixation) or (animal pictures–fixation) > (tool pictures – fixation) respectively, at a threshold of *z* > 2.3, with a cluster size probability of *p* < 0.05. The resulting clusters with an average tool picture preference (red) and an average animal picture preference (blue) for groups of 7- to 8-year-olds, 9- to 10-year-olds and adults are displayed on the standard *Freesurfer* surface in Fig. 2(top). Significant picture category-selective clusters of activation where located in approximately the same location as those previously reported in the adult-literature (see Appendix A, Table 2 for cluster statistics); At all ages, tool picture selective regions encompassed the bilateral medial fusiform gyrus (FFG), the bilateral middle temporal gyrus (MTG), a dorsal occipitoparietal cluster extending into the intraparietal sulcus encompassing the anterior intraparietal sulcus (AIP), the dorsal premotor cortex (dPMC) and left inferior frontal gyrus (IFG). Animal picture selective regions were located in the primary occipital cortex, and – more extensively in adults – the right FFG, and the right LOC just posterior to the region with a tool preference in the MTG. In line with findings by (Dekker et al., 2011) these activations where organised in a similar manner across all age groups. However, there were several areas where the amplitude of the category preference (tool pictures vs fixation – animal pictures vs fixation), varied linearly with age. These age-related changes involved both decreases and increases in the amplitude of category selective responses, depending on cortical area and picture category. See Appendix A, Fig. 1 and Table 3, for descriptions of areas where the amplitude of cortical category selectivity varied with age.

##### 3.3.1.2 Responses to animal and tool words per age group

In the activation maps in Fig. 2, clusters with a significant average category preference for printed words within each age group are depicted for tool words (yellow) and animal words (light green), and are indicated by arrows and labels (see Appendix A, Table 2 for cluster statistics). Considering that visual similarity and frequency of words were matched across category, it is not surprising that the differential neural responses to tool- and animal words are substantially smaller than those to tool- and animal pictures. Nevertheless, the group of adults showed a preference for tool names in a cluster in the left IFG/left dorsolateral prefrontal cortex (DLPFC), anterior – but adjacent – to an area with a preference for tool pictures in the IFG. Adults also showed a preference for tool names in the left LOC/MTG, in a region that partially overlapped with cortex with a preference for tool pictures. The group of 9- and 10-year-olds showed a preference for animal names in the left occipital pole, in a cluster that partially overlapped with a cortical area with a preference for animal pictures, but also with one with a preference for tool pictures. No regions with a category preference survived the statistical threshold in the group of 7- and 8-year-olds. A whole brain comparison of Tool vs Animal word processing across age did not reveal any significant group differences. In the next sections, we describe two types of ROI analyses (see Section 2) with greater detection power, in which tool versus animal word-processing is explored specifically within picture-category selective ROIs.

#### 3.3.2 Group average ROI analysis

##### 3.3.2.1 Are cortical areas with a category preference for pictures also engaged by the corresponding words?

To test whether the cortical areas with a selectivity for tool or animal pictures depicted in the activation maps in Fig. 2 showed a corresponding selectivity for tool or animal words, we extracted each individual’s BOLD-response to tool words (vs. fixation) and animal words (vs. fixation) from all voxels in age-specific clusters and computed each age group’s average category preference for words (tool words – animal words). The results are displayed in the bottom graphs in Fig. 2. Red bars indicate areas where subjects showed a significant preference for tool pictures *and* a corresponding stronger response to tool words. Similarly, blue bars indicate areas where the age group showed a significant preference for animal pictures *and* a corresponding preference for animal words. Grey bars indicate areas where the category preference for pictures and words did not correspond (e.g., a tool picture selective cluster with a stronger response to animal words). If printed words activate the same brain regions as their corresponding pictures, the category preference for animal and tool words should have the same direction as the local category preference for animal and tool pictures. In adults, this is clearly the case in all 6 ROIs. Overall, there was a significant category preference for tool and animals words in adult tool- and animal-picture selective cortical areas (*F*(1, 12) = 9.22, *p* = 0.010), and a trend towards an interaction effect of ROI × Category (*F*(5, 8) = 3.56, *p* = 0.055), indicating that category selectivity for words varied marginally across the 6 ROIs. In the group of 9- to 10-year-olds, the category preference for pictures and words was clearly less consistent, with corresponding response patterns in 4 out of 9 ROIs. There was no significant overall category preference (*F*(1, 9) = 0.647, *p* = 0.44), and no interaction of ROI × Category (*F*(8, 2) = 2.45, *p* = 0.33). Similarly, in 7- to 8-year-olds, 4 out of 7 regions showed a corresponding category preference for pictures and words and an ANOVA revealed no significant effects of Category (*F*(1, 10) = 0.025, *p* = 0.88) or Category × ROI (*F*(3.1, 31.1) = 1.74, *p* = 0.92. Due to the application of a statistical threshold, significant clusters from different age groups differ in number and areas of the brain they encompass (see Appendix A, Table 2). This limits the comparability of activation patterns in individual ROIs across age. To test if the age differences in category selectivity for animal versus tool words in these ROIs were significant, we therefore compared the response to tool and animal names averaged across all picture-selective ROIs. Crucially, this revealed that the picture-like responses to printed words were significantly more pronounced in adults than in children (Word Category × Age (adults vs children): *F*(1, 32) = 5.37, *p* = 0.027). Thus, while adults showed a clear picture-like activation in cortical sensory and motor regions when viewing written tool and animal names, words did not yet consistently engage the same areas as their corresponding pictures in children up to 10 years of age.

##### 3.3.2.2 Are cortical areas with a category preference for words also engaged by the corresponding pictures?

To test whether the brain areas with a preference for tool and animal words showed a similar response pattern for their corresponding pictures, we computed the relevant age group’s average category preference for pictures in these areas. In adults, both cortical regions with a preference for tool words also showed a significant preference for tool pictures (left IFG: *t*(12) = 4.02, *p* < 0.001, left FFG/MTG: *t*(12) = 2.5, *p* = 0.014). In the group of 9- to 10-year-olds the occipitoparietal area with a preference for animal pictures also showed a preference for animal words, although this effect did not reach statistical significance (*t*(12) = −1.05, *p* = n.s.). Thus, in adults and older children, brain regions with a significant category preference for tool or animal words also showed a category preference for the pictorial counterparts of those words, although the category preference for words was only significant in adults.

#### 3.3.3 Individual activation map ROI analysis

##### 3.3.3.1 Are voxels with a category preference for pictures also engaged by the corresponding words?

**Fig. 3:**
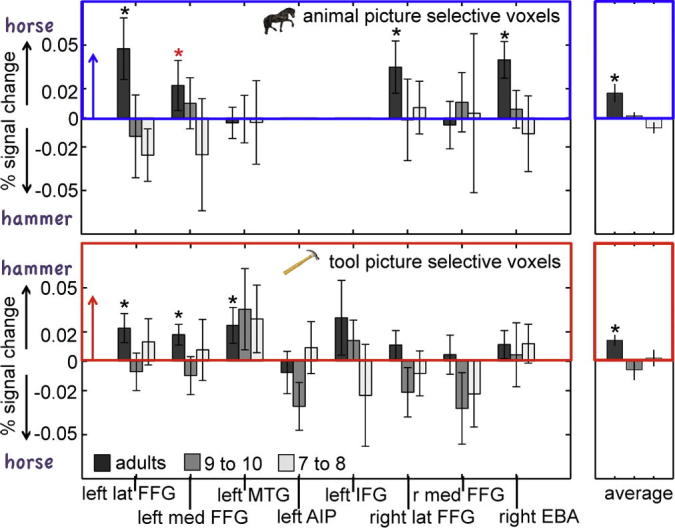
Dark grey bars: adults, medium grey bars: 9- to 10-year-olds, light grey bars: 7- to 8-year-olds. Top graph: Mean category preference for animal words (animal words vs fixation–tool words vs fixation), extracted from voxels with a significant (*p* < 0.05) preference for animal pictures in 6 a priori defined spherical ROIs. Bars pointing upwards into the blue frame indicate a corresponding category preference for animal words and pictures. Bottom graph: Mean category preference for tool words (tool words vs fixation–animal words vs fixation), extracted from voxels with a significant preference for tool pictures in 8 a priori-defined spherical ROIs. Bars pointing upwards into the red frame indicate a corresponding category preference for tool words and tool pictures. Significant category preferences for words (*p* < 0.05) are indicated by black stars, trends (*p* < 0.1) are indicated by red stars.

Fig. 3 displays the average category preference for words (tool words – animal words) in all animal picture selective voxels (top) and all tool picture selective voxels (bottom) within each spherical ROI and age group (see Section 2 for details on ROI selection, see Appendix C for % signal change in individual conditions relative to the fixation baseline). There were very few animal picture selective voxels in the left AIP and IFG so these regions were not included in the top graph, and were excluded from the analysis of animal-selective ROIs. ANOVA’s revealed that the picture-like category preference for words in these ROIs was significantly more pronounced in adults than in children (*Word Category* × *Age*, averaged across all ROIs: *F*(1, 32) = 5.21, *p* = 0.029), again indicating that picture-like category-selectivity for printed words changes with age. Specifically, areas with a preference for tool or animal pictures showed a similar preference for the corresponding printed word category in adults (*F*(1, 12) = 14.98 *p* = 0.002) while there was no evidence for such an overlap in either group of children (9- to 10-year olds: *F*(1, 9) = 0.128, *p* = 0.73; 7- to 8-year-olds: *F*(1, 10) = 0.051, *p* = 0.83). We also tested whether the local *direction* of the category preference for words and pictures in these ROIs was consistent in children, even though the average *amplitude* of the BOLD response reflected no such pattern. To this end, we counted the number of ROIs in each age group where the category preference for pictures and words was in the same direction, irrespective of whether this preference was significantly larger than zero. The chance of finding a corresponding category preference if there is no relationship between the category preference for words and pictures is 50%. In 117 out of 178 adult ROIs (14 ROIs × 13 subjects – 4 ROIs without tool or animal picture-selective voxels), the category preference for words corresponded with the local preference for tool or animal pictures. A sign test revealed that the probability of observing this proportion by chance is *p* < 0.0001. We therefore concluded that the category-selective response patterns for tools and animals in the adult brain were consistent across stimulus format. In contrast, in both groups of children, the proportion of ROIs with a corresponding category preference words and pictures was at chance level (9- to 10-year-olds: 64 out of 134 ROIs: *p* = 0.33, 7- to 8-year-olds: 72 out of 144 ROIs: *p* = 0.53), so, in both younger and older children, the local category preference for words and pictures was unrelated. Chi-square tests showed that adults had significantly higher proportions of areas with picture-like activations for words than the youngest and oldest group of children (overall age difference: *χ*^2^ = 12.56, df = 2, *p* = 0.002; adults vs 9- to 10-year-olds: *χ*^2^ = 10.134, df = 1, *p* = 0.001, adults vs 7- to 8-year-olds: *χ*^2^ = 8.13, df = 1, *p* = 0.004, 9- to 10-year-olds vs 7- to 8-year olds: *χ*^2^ = 1.39, df = 1, *p* = 0.71). We used Chi-square tests rather than ANOVA’s for this age comparison because the measure (whether ROIs show a corresponding category preference for words and for pictures or not) is categorical.

#### 3.3.4 Control analyses

##### 3.3.4.1 Do adults have more areas with picture-like responses to words than children matched on BOLD-related confounds?

In general, both examined BOLD-related confounds were higher in children than in adults. To test whether between-group differences in BOLD-related confounds could explain the absence of sensorimotor activations for words in children, we compared the consistency of category preferences across stimulus format in subgroups of 9 adults and 9 children matched on these confounds (see Section 2 and Appendix B, Table 1). Confound-matched adults showed significantly more areas with a corresponding category preference for words and pictures than confound-matched children (*χ*^2^ = 5.54, df = 1, *p* = 0.019). Moreover, sign tests revealed that the number of areas with a corresponding preference for tool or animal words and pictures was higher than chance-level in adults (*p* < 0.001) but not in children with similar levels of BOLD confounds (*p* = 0.235). Thus, the absence of sensorimotor activation when children read familiar words, was not due to BOLD-related confounds.

## 4 Discussion

Embodiment theories and research supporting these theories for adults, suggest that printed word meaning is at least partially represented in cortical regions that also process sensorimotor properties of the object categories described by these words (Barsalou, 2008, Fischer and Zwaan, 2008, Pulvermueller, 2013). During reading training, children learn to extract semantic information from abstract words shapes. It is unclear how and when during this process, printed words start activating cortical sensorimotor representations associated with meaning processing. We therefore investigated when printed tool and animal words start engaging the same category-specific cortical regions as the pictures that they describe (e.g., for tools: dorsal motor cortex involved in grasping and occipitotemporal cortex processing tool motion and shape, for animals: occipital regions processing biological motion and faces). We did this by measuring BOLD-responses to tool versus animal pictures and printed tool versus animal names in the brains of 7- to 8-year-olds, 9- to 10-year-olds and adults during a one-back categorisation task.

We first established in a whole brain analysis, that all participants showed clear differential cortical specialisation for tool versus animal pictures. Tool picture-selective regions encompassed the bilateral medial FFG, the bilateral MTG, the dorsal occipitoparietal cortex extending into AIP, the dPMC, and the left IFG. Animal picture selective regions encompassed the primary occipital cortex, and – more extensively in adults – the right FFG, and the right LOC. The cortical organisation of tool and animal picture selective areas was largely consistent across age, although there were some age-related decreases and increases in the extent of picture category preference depending on object type and brain area. So, even in the brains of the youngest group of children category-specific sensorimotor networks for tool and animal categories were in place.

In a second whole brain analysis, we explored for each age group, which brain areas showed category-selective responses for printed tool versus animal words. We also checked if these areas showed the same category-selective responses for the words’ corresponding pictures. In adults, two areas were found to be selective for tool words as well as tool pictures. One of these areas was located in left middle temporal gyrus, associated with tool motion processing (Beauchamp, Lee, Haxby, & Martin, 2002) and the other one was located in the inferior frontal gyrus, involved in selection and planning of tool-related actions (Fagg and Arbib, 1998, Gallese et al., 1994). There were no brain areas with a category preference for tool or animal words in 7 to 8-year-olds. While the group average activation map of children aged 9–10 years contained one occipital area that was selective for animal words, there was no significant animal picture selective BOLD-response in this brain area. So in childhood, we identified no brain regions that were selective for tool or animal words and that also showed corresponding category-selectivity for pictures. At the whole brain level, these age-differences in word category processing did not reach statistical significance.

To explore BOLD-responses to printed tool versus animal words in category-selective sensorimotor areas of the cortex directly, we performed two region-of-interest analyses. As explained in the Section 2, ROIs for these analyses were made up of (i) animal or tool picture selective clusters in group-average activation maps and (ii) animal or tool picture selective voxels within pre-defined locations in individual activation maps. Both analyses showed that in adults, ROIs across the sensorimotor cortex with a selective response to tool or animal pictures, tended to show a similar category preference for these picture’s printed names. In contrast, the directions of category-selective response patterns for tool versus animal pictures and tool versus animal names were entirely unrelated in the 7 to 8-year-old and 9 to 10-year-old sensorimotor cortex. Crucially, statistical tests comparing BOLD-responses derived from type (i) and (ii) ROIs across age, revealed that category-selective responses to printed tool and animal names were significantly more pronounced in the adult cortex than in the child cortex. These results can thus not simply be ascribed to greater increases in BOLD activity in adults than in children.

In subgroups of adults and children matched on scan-to-scan motion and residual noise in the GLM, adults still showed significantly more ROIs with corresponding category-selectivity for pictures and their printed names than children. Therefore, the age-differences reported here are unlikely to be driven by BOLD-related confounds. It is also unlikely that they are caused by reduced attention or poorer task-performance in children, because accuracy on the one-back task in the scanner was far above chance level and equivalently high across all ages and conditions.

In adults, areas in the cortex that were category-selective for tool versus animal pictures thus clearly showed corresponding category-selectivity for the words describing those pictures in our one-back matching task. This is consistent with the notion that “embodied” category knowledge is activated automatically during reading in the mature cortex (Pulvermueller, 2013). Based on picture-word priming effects in young readers that suggest automatic co-activation of semantic representation across formats (Ehri, 1976, Rosinski, 1977, Rosinski et al., 1975), we expected spontaneous picture-like BOLD-responses to printed words to emerge early in reading training. However, we found the opposite, namely that it takes years of training and highly expert reading skills, before familiar printed words give rise to automatic picture-like activations in the cortices of developing readers.

Why does sensorimotor cortex engagement during printed word processing take so long to develop? One possibility is that children performed the matching task in the scanner solely by focussing on word shape, without any processing of word content (i.e., without automatic reading). Whilst we cannot fully exclude this possibility because we collected no reading measures in the scanner, we believe this explanation is highly unlikely. Firstly, because high task performance indicates that children were paying close attention to the stimuli on the screen, and secondly because reading measures collected before scanning show that they could read the words well within their presentation time – especially the older fluent readers. Thirdly and most importantly, we believe it is unlikely that children were able to refrain entirely from reading because previous studies have shown that printed words induce semantic priming (and interference) effects in children with similar ages and reading expertise as the youngest subjects in our study, even if word primes are ignored or presented briefly (Chapman et al., 1994, Ehri, 1976, Plaut and Booth, 2000, Rosinski, 1977, Rosinski et al., 1975, Simpson and Foster, 1986, Simpson and Lorsbach, 1983). This strongly suggests that viewing single printed familiar words can automatically evoke meaning processing in childhood readers, even during visual tasks and when their reading fluency is relatively poor.

A more likely possibility is therefore, that the neural mechanisms that translate word shape into sensorimotor meaning are still not fully developed by the 11th year of life. The occipito-temporal cortex only starts showing adult-like sensitivity for word forms at around the 14th year of life (Ben-Shachar, Dougherty, Deutsch, & Wandell, 2011), when measures of reading fluency also reach adult levels (Wechsler, 2001). In line with the Interactive Specialisation theory of brain development (Johnson, 2011), this process likely reflects increasing neural sensitivity to word shapes locally, but might also involve the improvement of connectivity with remote sensorimotor representations distributed across the cortex. Support for this Interactive Specialisation framework comes from resting state fMRI studies showing increasing functional connectivity between various motor and occipitotemporal cortex areas associated with reading (Koyama et al., 2011), and more general decreases in local connectivity and increases in long-range connectivity across the brain until well into the teenage years (Dosenbach et al., 2010, Fair et al., 2007). In adults, sensorimotor cortex responses to printed words depend heavily on task-context (Mahon and Caramazza, 2008, Pulvermueller, 2013, Willems and Casasanto, 2011). For example, Devlin et al. (2005) showed that category-selective activation for printed tool and animal names in the fusiform gyrus was more pronounced during categorising (man-made or natural?), than during perceptual judging of word-length (longer or shorted than comparison line?). This task-dependency might be even stronger during childhood if communication between visual word form areas and sensorimotor representations of word meaning is less direct or efficient. Expert adult readers may spontaneously picture the sensorimotor properties of objects they are reading about, thus activating for example brain areas involved in action planning for tool names and areas involved in body and face processing for animal names. Children, instead, may activate knowledge with weaker links to sensorimotor experience when reading words, such as object names or functions, resulting in reduced differential activity for printed tool- and animal names in their picture category-selective cortex.

An interesting next step would be to explore how sensorimotor cortex engagement during explicit word comprehension tasks changes across age. This will help disentangle further how word processing strategies and developmental constraints contribute to reduced activation of “embodied” category representations for printed words in childhood. Due to sluggishness of the BOLD-response, fMRI is not ideal for establishing if sensorimotor cortex responses in word comprehension at different ages result from slow, deliberate word meaning processing or the rapid automatic process reported for skilled adult readers (Hauk et al., 2008, Kiefer et al., 2008). This issue can be addressed in the future by complementing fMRI measures of sensorimotor cortex activation high in spatial resolution, with EEG measures high in temporal resolution. For example, by comparing the time course of gamma-band de-synchronisation over the motor cortex (an index of motor cortex activation) during tool versus animal name reading across age.

In conclusion, children and adults both showed clear differential cortical specialization when matching tool and animal pictures on basic-level category. However, while adults co-activated the same animal and tool picture-selective cortical regions when performing this task with the pictures’ written names, children did not. This was despite the fact that all children could read and comprehend all names in the experiment and despite substantial reading proficiency in the older children. This gradual emergence of neural responses thought to play a crucial role in printed word comprehension and its development, suggests that until a relatively late age and advanced level of reading proficiency, children do not spontaneously experience the sensorimotor meaning of single printed words they read. These results form a first step towards understanding how printed word meaning becomes “embodied” as children learn to link word shapes to word meanings.

## Funding

This work was funded by a European Commission grant MEST-CT-2005-020725 (CBCD) and ITN-CT-2011-28940 (ACT). TMD was partly funded by an Economic & Social Research Council grant RES-061-25-0523, DM is supported in part by a Royal Society Wolfson Research Merit Award, MHJ is funded by the UK Medical Research Council, G0701484, and MIS is funded by a National Institutes of Health grant R01 MH 081990 and a Royal Society Wolfson Research Merit Award.
