# Supplementary material for: Picturing words? Sensorimotor cortex activation for printed words in child and adult readers
Source: Brain Lang. 2014 Dec;139:58–67. doi: 10.1016/j.bandl.2014.09.009 (PMC4271739; doi:10.1016/j.bandl.2014.09.009)
Supplement: Supplementary data 1 [file mmc1.pdf]

Appendix A

Table A.1

Animals	UK wrd freq	Tools	UK wrd freq
**Bear**	19	Cup	59
**Bird**	42	Fork	12
**Horse**	85	Knife	35
**Lamb**	15	Glasses	51
**Penguin**	4	Paintbrush	1
**Butterfly**	5	Pan	22
**Tiger**	4	Pen	19
**Cat**	41	Pencil	15
**Cow**	22	Broom	6
**Turtle**	1	Brush	13
**Deer**	6	Comb	10
**Dog**	70	Rubber	25
**Duck**	4	Scissors	4
**Elephant**	5	Spade	3
**Fish**	83	Teapot	3
**Zebra**	1	Hammer	10
**Frog**	4	Toothbrush	2
**Giraffe**	1	Bottle	82
**Pig**	18	Violin	4
**Rabbit**	11	Key	71
**MEAN FREQ:**	**22.1**	**MEAN FREQ:**	**22.35**

Table A.1. All printed words included in the study. Only words that are acquired before the age of 7 were included (MRC database Max Planck: http://www.psy.uwa.edu.au/mrcdatabase/uwa_mrc.htm). The tool- and animal words were matched on average number letters, syllables and written word frequency as described in the British version of the Celex2 database (Baayen, Piepenbrock, Gulikers, 1996). The written word frequencies are reported. Examples of word stimuli and related pictures are displayed in Figure 1.

Table A.2

Category preference for pictures or words:	Nvox	Z_{max}	X_{mm}	Y_{mm}	Z_{mm}
**Adults, tools > animals**					
Pictures:					
Right occipitoparietal cortex, superior and inferior parietal Cortex, medial and lateral FFG, LOC, MTG	2770	3.8	26	-70	30
Left occipitoparietal cortex & superior and inferior parietal cortex (incl. AIP)	2315	4.15	-24	-76	46
Left medial and lateral FFG, LOC, MTG	1472	4.48	-40	-66	-6
Left inferior frontal gyrus	1001	3.66	-52	16	14
Right superior frontal /paracingulate gyrus	414	3.28	14	24	54
Words:					
Left IFG	313	3.28	-42	36	6
Left lateral FFG, LOC, MTG	301	3.23	-46	-48	2
**9 to 10, tools > animals**					
Pictures:					
Left occipitoparietal cortex, superior and inferior parietal cortex (incl. AIP)	2375	3.89	-28	-82	14
Right medial and lateral FFG, LOC, MTG	1366	3.94	50	-60	-8
Right occipitoparietal cortex & superior parietal cortex	1243	3.98	24	-68	56
Left lateral FFG, LOC, MTG	691	4.52	-48	-64	-12
Anterior IFG	402	3.15	-38	26	18
Medial FFG	352	3.71	-26	-52	-14
Precentral Gyrus, posterior IFG	310	3.26	-44	6	28
Words:					
No significant clusters	-	-	-	-	-
**7 to 8, tools > animals**					
Pictures:					
Left occipitoparietal cortex & superior and inferior parietal cortex (incl. AIP)	5590	4.61	-36	-52	52
Right occipitoparietal cortex & superior and inferior parietal cortex	3849	4.16	30	-78	16
Left medial and lateral FFG, LOC, MTG	1613	4.5	-48	-64	-8
Right lateral FFG, LOC, MTG	666	4.3	52	-60	-18
Left posterior IFG	665	3.6	-50	6	26
Left precentral Gyrus	410	3.64	-24	-12	50
Words:					
No significant clusters	-	-	-	-	-
**Adults, animals > tools**					
Pictures:					
Bilateral Occipital cortex and posterior FFG, right LOC, left and right lateral FFG (incl. right FFA)	9648	5.28	16	-90	-10
Words:					
No significant clusters	-	-	-	-	-
**9 to 10, animals > tools**					
Pictures:					
Bilateral Occipital cortex and posterior FFG	6643	5.52	18	-94	8
Right LOC	518	4.5	46	-76	-2
Words:					
Left calcarine sulcus	512	3.03	-20	-88	10
**7 to 8, animals > tools**					
Pictures:					
Bilateral Occipital cortex and posterior FFG, right LOC	7566	5.11	18	-94	6
Words:					
No significant clusters	-	-	-	-	-

Table A.2. Cluster statistics of all regions displayed in Figure 2 (main text) with a significant average preference for tool pictures, animal pictures, tool words and animal words in 11 7- to 8-year-olds, 10 9- to 10-year-olds and 13 adults. All cluster surpassed a threshold of z=2.3, $p_{cluster} < 0.05$, in a whole brain comparison within each age group.

Figure A.1

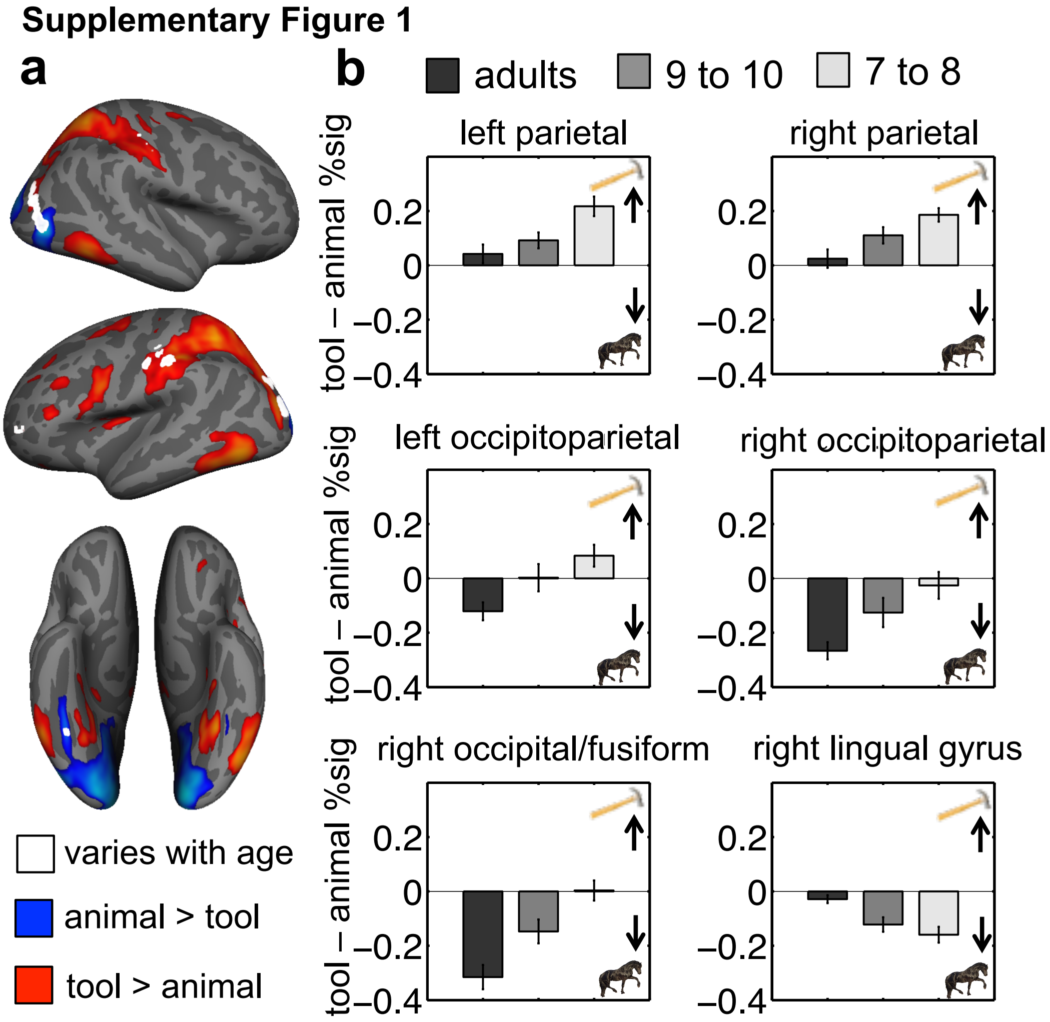

Figure A.1 Areas where the amplitude of picture category selectivity (tool vs fixation – animal vs fixation) varied linearly with age (z=2.3, $p_{cluster} < 0.05$) in a whole brain analysis are displayed (white) on top of a group average activation map (n=34, all subjects) with animal picture selective regions (blue) and tool picture selective regions (red). B) Graphs visualise the patterns of age-related changes in the white

clusters. Dark grey bars: adults, medium grey bars: 9- to 10-year-olds, light grey bars: 7- to 8-year-olds. The amplitude of tool picture selectivity in the left and right superior- and intraparietal cortex decreased with age. Selectivity for animal pictures in the right fusiform gyrus, around human FFA and in the right and left occipitoparietal cortex emerged with age, in line with previous reports of protracted specialisation for human faces in these regions. The amplitude of animal picture selectivity in the lingual gyrus decreased with age. Thus, there were age-related decreases and increases in the amplitude of animal- and tool picture selectivity depending on cortical location and picture category. In contrast, Dekker et al. (2011) reported developmental consistency in tool picture selectivity 6 years onwards. The different findings might be explained by (a) different depth of object property processing in the current categorisation task compared to the passive viewing task used by Dekker et al. (2011) (b) different choice of baseline (scrambled objects vs fixation).

Table A.3

**Age (N=34) correlates with tool pictures > animal pictures:**	Nvox	Z_{max}	X_{mm}	Y_{mm}	Z_{mm}
left frontal pole	336	3.69	-24	70	-8
right lingual gyrus	308	3.64	12	-76	-2
right Occipitoparietal	825	4.45	34	-74	10
left superior and inferior parietal	669	3.59	-36	-58	64
left Occipitoparietal	559	2.95	-18	-104	14
right superior and inferior parietal	374	1.77	28	-58	70
right Occipital/Fusiform	343	1.55	44	-56	-24

Table A.3. Cluster statistics of regions where the category preference for tools or animals (contrast: tool pictures vs fixation – animal pictures vs fixation), changed linearly with age from childhood to adulthood. All cluster surpassed a threshold of $z=2.3$, $p_{cluster} < 0.05$ and are depicted in Fig. S2 (white).
