# Supplementary material for: Picturing words? Sensorimotor cortex activation for printed words in child and adult readers
Source: Brain Lang. 2014 Dec;139:58–67. doi: 10.1016/j.bandl.2014.09.009 (PMC4271739; doi:10.1016/j.bandl.2014.09.009)
Supplement: Supplementary data 2 [file mmc2.pdf]

Appendix B
Table B.1

	ALL SUBJECTS		MATCHED SUBJECTS	
Subject details:	Children<br><br>N = 21<br>Ages: 7.08 -10.8<br>Mean Age: 8.9	Adults<br><br>N = 13<br>Ages: 21.4 – 42.6<br>Mean Age: 28.1	Children<br><br>N = 9<br>Ages: 7.08 -10.8<br>Mean Age: 8.9	Adults<br><br>N = 9<br>Ages: 21.4 – 42.6<br>Mean Age: 28.9
Motion:				
ΔD:	0.058 ± 0.03* ↔	0.030 ± 0.01	0.036 ± 0.01 ↔	0.033 ± 0.02
ΔR:	$0.42\times10^{-3} \pm 2\times10^{-4}$ * ↔	$0.18\times10^{-3} \times 8\times10^{-5}$	$0.27\times10^{-3} \pm 7\times10^{-5}$ * ↔	$0.21\times10^{-3} \times 8\times10^{-5}$
%Res	0.012 ± 0.003* ↔	0.0096 ± 0.001	0.010 ± 0.001 ↔	0.0097 ± 0.001

Table B.1: Mean scan-to-scan Euclidean translation distance in mm (ΔD), the mean scan to scan rotation in radians(ΔR) and the percentage of residual noise in the signal after fitting the GLM (%Res) for all children and all adults (left) and for subgroups of children and adults matched on these potential confounds (right). Black stars behind child values indicate that the difference between adults and children is significant ($p < 0.01$). Red stars indicate non-significant trends ($0.05 < p < 0.1$). There were significant differences in all three measures when comparing all children and adults who took part in the study; scan to scan translation distance: $F(1,31)= 7.6$, $p=0.01$; scan to scan rotation: $F(1,31)= 15.5$, $p<0.001$; residual variance: $F(1,31)= 3.03$, $p=0.01$. In the subgroup of 9 adults and 9 children matched on these potential confounds, there were no significant differences in these three measures: scan to scan translation: $F(1,16)= 0.002$, $p =0.97$, scan to scan rotation: $F(1,16)= 3.2$, $p = 0.09$, residual noise variance: $F(1,16) = 1.1$, $p = 0.3$.
