# Supplementary material for: Picturing words? Sensorimotor cortex activation for printed words in child and adult readers
Source: Brain Lang. 2014 Dec;139:58–67. doi: 10.1016/j.bandl.2014.09.009 (PMC4271739; doi:10.1016/j.bandl.2014.09.009)
Supplement: Supplementary data 3 [file mmc3.pdf]

Appendix C

Graphs display mean % signal change and standard errors, for tool- (red) and animal words (blue) verus fixation, within picture category selective voxels in spherical ROIs of each group (differential responses computed within subjects are displayed in Figure 3).

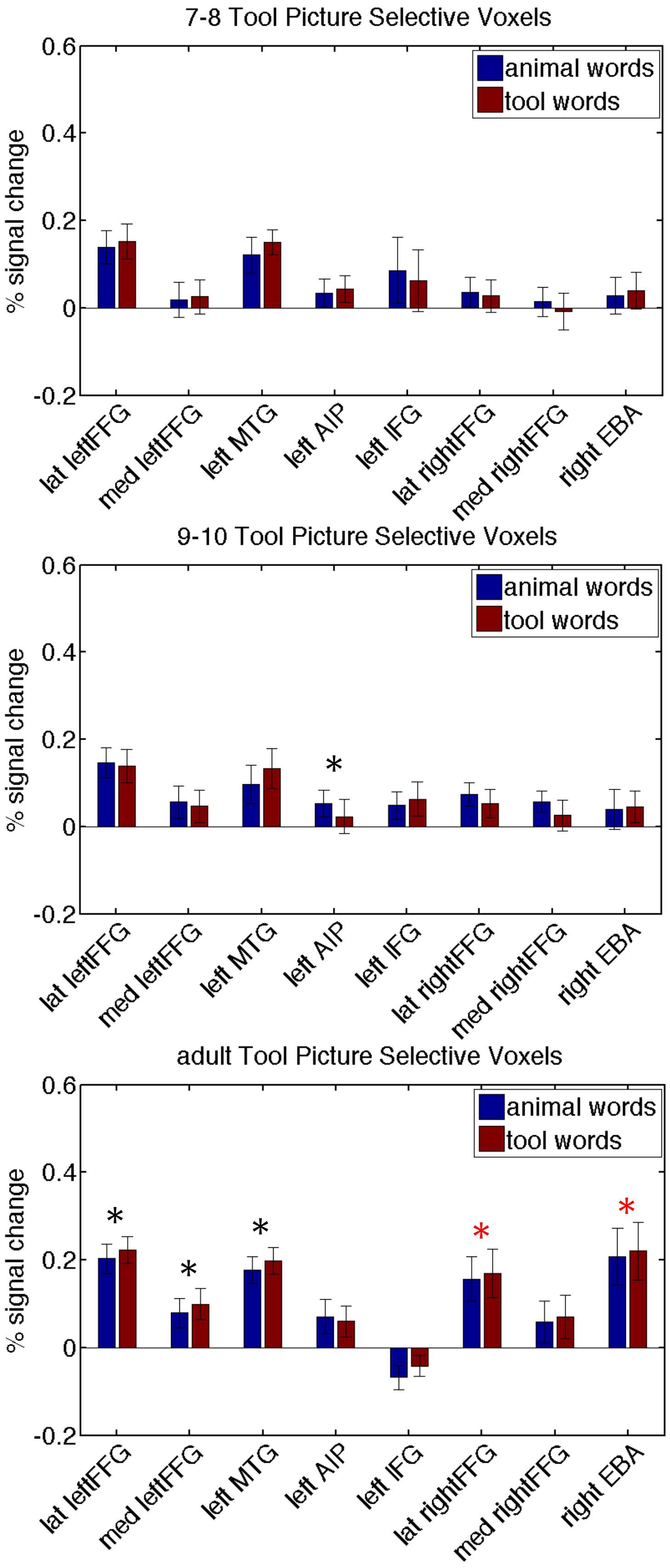

* = $p<0.05$

* = $p<0.1$

in paired t-test

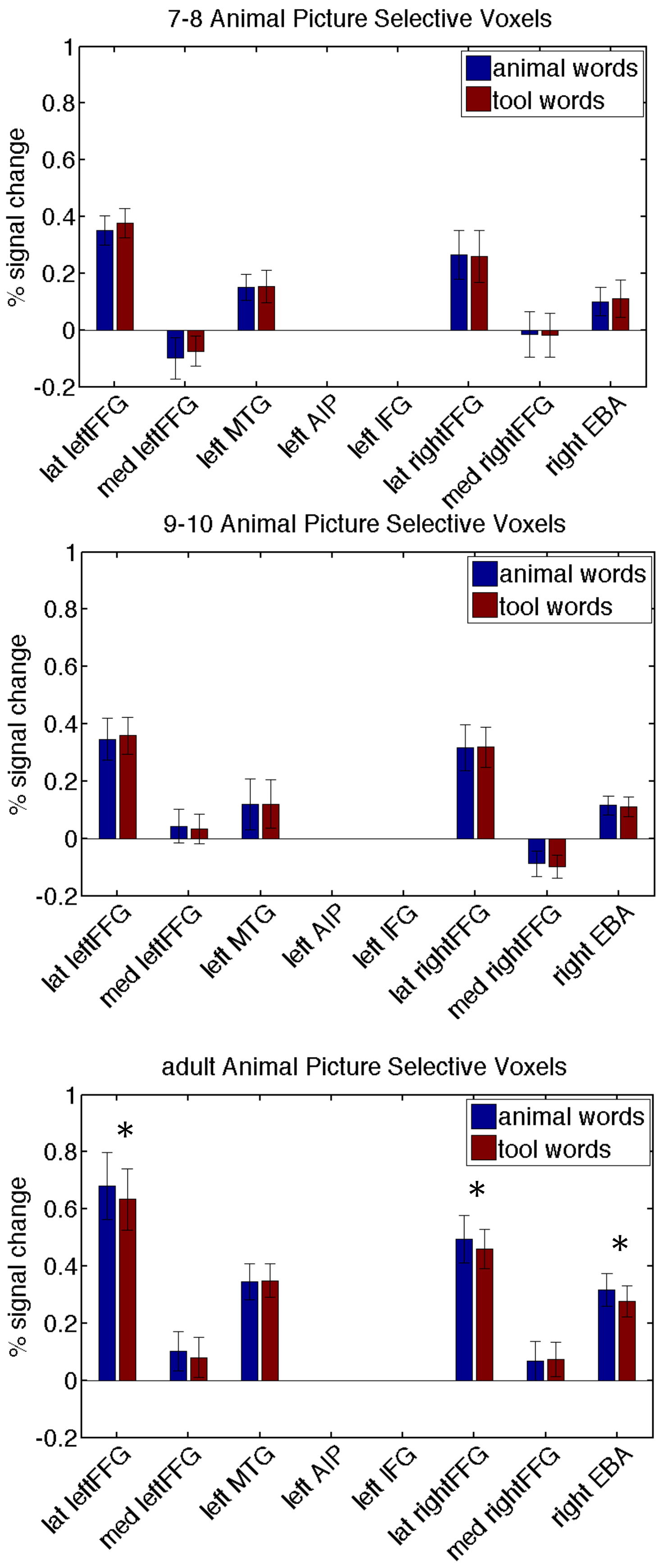
7-8 Animal Picture Selective Voxels
% signal change
1
0.8
0.6
0.4
0.2
0
-0.2
animal words
tool words
lat leftFFG
med leftFFG
left MTG
left AIP
left IFG
lat rightFFG
med rightFFG
right EBA
9-10 Animal Picture Selective Voxels
% signal change
1
0.8
0.6
0.4
0.2
0
-0.2
animal words
tool words
lat leftFFG
med leftFFG
left MTG
left AIP
left IFG
lat rightFFG
med rightFFG
right EBA
adult Animal Picture Selective Voxels
% signal change
1
0.8
0.6
0.4
0.2
0
-0.2
animal words
tool words
*
*
*
lat leftFFG
med leftFFG
left MTG
left AIP
left IFG
lat rightFFG
med rightFFG
right EBA
